# Efficacy and Safety of Combined Treatment with Traditional Herbal Medicine and Western Medicine for Children with Pertussis-like Syndrome: Systematic Review and Meta-Analysis

**DOI:** 10.3390/healthcare13101131

**Published:** 2025-05-13

**Authors:** Ji-U Choi, Young-Shin Shim, Eun-Jin Kim, Sang Yeon Min

**Affiliations:** 1 Department of Pediatrics of Korean Medicine, Graduate School, Dongguk University, Seoul 04620, Republic of Korea; choizwoo@naver.com (J.-U.C.); simyoungshin@naver.com (Y.-S.S.); 2 Department of Pediatrics of Korean Medicine, Korean Medicine Hospital, Dongguk University Bundang Medical Center, Seongnam-si 13601, Republic of Korea; utopialimpid@naver.com; 3 Department of Pediatrics of Korean Medicine, Korean Medicine Hospital, Dongguk University Ilsan Medical Center, Goyang-si 10326, Republic of Korea

**Keywords:** herbal medicine, complementary treatment, whooping cough, meta-analysis

## Abstract

**Background/Objectives**: Pertussis-like syndrome (PLS) presents symptoms similar to whooping cough but without *Bordetella pertussis* detection. This study assessed the efficacy and safety of combined treatment herbal and Western medicine (HM and WM, respectively) for PLS. **Methods:** Eleven English, Chinese, Korean, and Japanese databases were searched until 1 December 2024. Randomized clinical trials (RCTs) that compared HM with WM versus WM alone in children with PLS were included. Independent searches and risk-of-bias analyses were conducted. Random-effects and fixed-effects models were utilized. Dichotomous outcomes are presented as the risk ratio (RR) with 95% confidence interval (CI), and continuous outcomes as either the standard mean difference (SMD) or mean difference (MD) with 95% CI. **Results:** A total of 23 RCTs (performed in China) with 1938 participants were included. The meta-analysis showed that HM with WM is more effective than WM in improving the total effective rate [*n* = 1888; RR = 1.20; 95% CI: 1.16–1.24; *p* < 0.001], reducing the disappearance time of main symptoms (especially spastic cough) [*n* = 815; MD = −3.31; 95% CI: −3.51–−3.11; *p* < 0.001], shortening the recovery time of routine blood parameters to the normal range [*n* = 472; MD = −2.79; 95% CI: −3.06–−2.52; *p* < 0.001], and decreasing hospitalization duration [*n* = 703; MD = −2.61; 95% CI: −2.85–−2.38; *p* < 0.001]. Only mild adverse events were reported, with a lower occurrence rate in HM with WM cohorts than in WM cohorts. The quality of evidence ranged from moderate to very low. **Conclusions**: HM combined with WM is effective and safe for PLS in children, offering a potential alternative for symptom relief.

## 1. Introduction

Whooping cough (pertussis) is a type of acute respiratory disease induced by *Bordetella pertussis* (*B. pertussis*) infection and is among the top 10 fatal infections in children [1]. When pertussis infection is suspected, clinical history and symptoms are evaluated. To confirm the diagnosis, laboratory tests, such as white blood cell count, absolute lymphocyte count, bacterial culture, polymerase chain reaction, and chest X-ray, should be conducted [2]. Pertussis is a global infectious disease that requires reporting of cases to the government. According to the World Health Organization (WHO), global pertussis incidence rates were 4.6–9.9 during 2020–2022. These rates surged to 22.7 and 35.7 per 1,000,000 total population during 2023 and 2024, respectively [3]. In particular, the pertussis resurgence rate in Europe in 2023 was approximately 14 times higher than that in 2022 [3]. The incidence rates were also higher in 2024 than in the same period in 2023 in various countries, including the United States (six times), South Korea (164 times), and China (12 times) [4,5,6].

If clinical signs and test results do not confirm pertussis, the condition is referred to as a pertussis-like syndrome (PLS). PLS is reported at any age but is more prevalent in children. PLS can be particularly challenging for children and their parents because its symptoms often disrupt quality of life and cause sleep disorders [7]. In one study, only 19.8% of children with similar symptoms tested positive for *B. pertussis* [8]. Consequently, the incidence of pertussis has increased rapidly, indicating that PLS should be treated with caution.

Whooping cough has an incubation period of 5–21 days. The catarrhal stage (1–2 weeks) is characterized by upper respiratory symptoms such as rhinorrhea, excessive tearing, and mild cough. The paroxysmal stage (1–6 weeks) is characterized by severe spastic cough followed by post-tussive vomiting. In the convalescent stage (lasting for 2–4 weeks), paroxysmal cough may recur due to infections [2]. However, *B. pertussis* infection is not the only cause of these clinical symptoms. Other pathogens, including adenovirus, influenza virus, and *Mycoplasma* species, can cause similar symptoms, collectively termed PLS, making differential diagnosis challenging due to the lack of distinctive clinical features [9].

In Western medicine (WM), whooping cough (and PLS) is generally treated with antibiotics, such as erythromycin and azithromycin [10]. Erythromycin and azithromycin are also used to treat other respiratory diseases, such as diffuse panbronchiolitis, bronchiectasis, rhinosinusitis, and cystic fibrosis, as inflammation and immunity modulators [11]. The limitation of antibiotic use is that it does not dramatically relieve the clinical symptoms of the illness, such as spastic cough, even though the treatment is effective in eliminating pathogens [12]. This has prompted interest in herbal medicines (HMs) as potential alternatives to WM for symptom relief. Previous studies have demonstrated the efficacy of HM in managing respiratory diseases. For example, a combination of HMs including *Glycyrrhizae Radix et Rhizoma* and *Pinelliae Rhizoma* improved lung function in patients with asthma, which is characterized by spastic cough but not an infectious disease, and also reduced the dosage of WMs [13]. HMs (such as Zhisou-san) are effective in decreasing cough severity and recurrence rates in nonspecific chronic coughs, and *Glycyrrhizae Radix et Rhizoma*, *Pinelliae Tuber*, *Platycodonis Radix*, and *Armeniacae Semen* are frequently used herbs [14]. Since HM use can relieve symptoms of respiratory diseases that are difficult to manage using only WM, a combination of HM and WM in PLS may also be effective.

No meta-analysis or systematic review has addressed the use of HMs for PLS. This study aimed to evaluate the efficacy and safety of the combination of HM and WM for PLS.

## 2. Materials and Methods

### 2.1. Protocol and Registration

This study followed the Preferred Reporting Items for Systematic Reviews and Meta-Analyses (PRISMA) 2020 guidelines [15] (Table S1). The study protocol was registered in PROSPERO (ID: CRD42024561529), and modified to specify the outcome indicators.

### 2.2. Eligibility Criteria

#### 2.2.1. Types of Studies

RCTs evaluating HM for PLS in children and adolescents were included. Non-RCTs, case reports, animal studies, gray literature, surveys, and review articles were excluded.

#### 2.2.2. Types of Participants

Participants included children and adolescents aged <18 years diagnosed with PLS. Those diagnosed with *B. pertussis* infection were excluded.

#### 2.2.3. Types of Interventions

The experimental group received oral HM, with no restrictions on the number of herb ingredients, formulations (such as powder, granules, or decoctions), dosages, or duration. Conventional treatments, including WMs, such as azithromycin and erythromycin, were considered only if the control group received identical treatments.

#### 2.2.4. Types of Comparisons

If conventional treatments were administered to the experimental group, equivalent treatments were provided to the control group.

#### 2.2.5. Types of Outcome Measurements

The main outcome was the total effective rate (TER). Secondary outcomes included the disappearance time of primary symptoms, such as spastic cough, the recovery time of blood routine parameters to normal range, hospitalization duration, and adverse events.

### 2.3. Information Sources and Search Strategy

Eleven databases were searched until 1 December 2024, without limitations on country, language, and publication year. The databases included three English-language sources (PubMed, Excerpta Medica Database, and the Cochrane Central Register of Controlled Trials), three Chinese databases (China National Knowledge Infrastructure, VIP, and Wanfang Database), four Korean databases (Oriental Medicine Advanced Searching Integrated System, Korean Studies Information Service System, Korea Citation Index, and Research Information Sharing Service), and one Japanese database (CiNii). The main terms for searching included “whooping cough”, “pertussis-like syndrome”, and “herbal medicine”, which were tailored to each database’s language requirements. More specific search strategies and results are included (Table S2).

### 2.4. Study Selection and Data Extraction

#### 2.4.1. Study Selection

Two reviewers (J.U.C. and Y.S.S.) independently screened titles and abstracts, and next then assessed full-text articles based on the inclusion and exclusion criteria. Any disagreements were resolved through discussion, and an additional reviewer (E.J.K.) made the final decision if there was no agreement.

#### 2.4.2. Data Extraction

Two reviewers (J.U.C. and Y.S.S.) independently extracted information from the included studies. Any discrepancies were resolved through discussion among all reviewers. The missing information was resolved by contacting the corresponding author via email. Extracted data included first author, publication year, study location, sample size, duration of disease, total treatment period, participant characteristics, such as gender and age distribution, treatment interventions and comparisons, HM composition, outcome measurements, adverse events, and data for risk of bias assessment.

### 2.5. Statistical Analysis

Review Manager (RevMan) software (version 5.4, Cochrane Collaboration, London, UK) was used for performing the meta-analysis. Both random-effects and fixed-effects models were used. For dichotomous outcomes, the risk ratio (RR) with 95% confidence intervals (CIs) was applied, whereas for continuous outcomes, either the standard mean difference (SMD) or mean difference (MD) with 95% CIs was applied.

#### 2.5.1. Assessment of Heterogeneity

Heterogeneity among included studies was assessed by using the Higgins *I^2^* index [16]. When *I^2^* was ≥50%, it indicates significant heterogeneity, and a random-effects model was applied. When *I^2^* was <50%, indicating low heterogeneity, a fixed-effects model was applied.

#### 2.5.2. Assessment of Reporting Bias

Publication bias was assessed when >10 studies were included for a specific outcome. Funnel plots were presented to visualize bias. If asymmetry suggested potential bias, Egger’s regression test, trim-and-fill, and Rosenthal fail-safe N method were performed.

#### 2.5.3. Subgroup and Sensitivity Analysis

Due to the variability in HM composition, subgroup analyses were performed based on specific HM formulations. Sensitivity analysis was performed for the outcomes when data from at least 10 studies were available.

### 2.6. Quality Assessment

Two reviewers (J.U.C. and Y.S.S.) independently assessed the quality of included studies using the Cochrane Risk of Bias (RoB 2) tool, categorizing studies as having low risk of bias, some concerns regarding bias, or high risk of bias [17]. The assessment included five domains: randomization process, deviations from intended interventions, missing outcome data, measurement of outcomes, and selection of reported results. Any discrepancies between reviewers were resolved through discussions, and if there was no agreement, a third reviewer (E.J.K.) made the final decision.

The quality of evidence was assessed using the Grading of Recommendations, Assessment, Development, and Evaluation (GRADE) framework (http://gradepro.org (accessed on 1 February 2025)). The evaluation considered risk of bias, inconsistency, indirection, imprecision, and publication bias. The GRADE system categorized the quality of evidence as very low, low, moderate, and high.

## 3. Results

### 3.1. Study Selection

**Figure 1:**
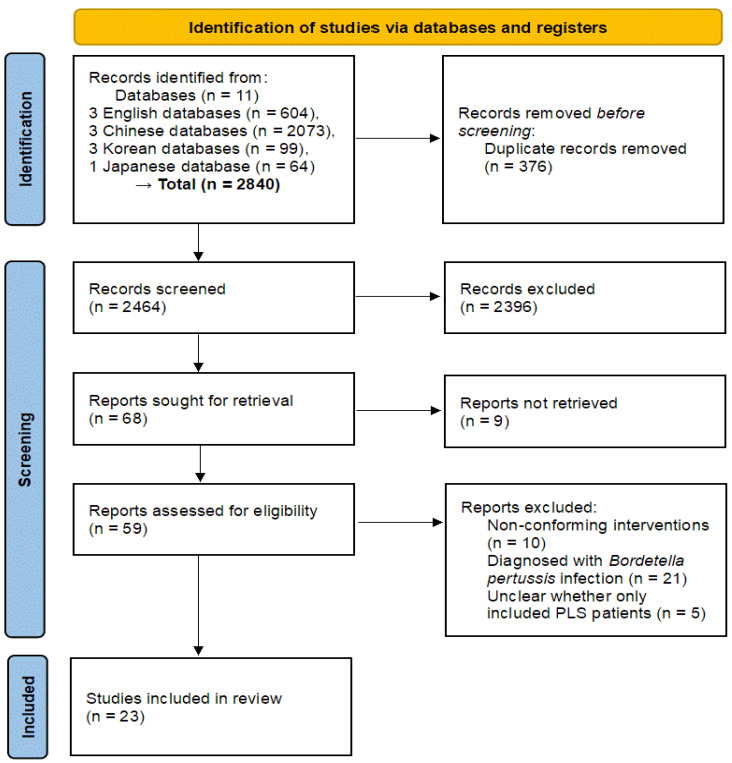
PRISMA 2020 flow diagram. PLS, pertussis-like syndrome.

After applying the search strategy to each database, a total of 2840 records were retrieved. These consisted of 604 from English databases, 2073 from Chinese databases, 99 from Korean databases, and 64 from the Japanese database. Following deduplication, 2464 studies were left. After reviewing titles and abstracts, 68 studies were selected for further evaluation; however, 9 studies were inaccessible. All the 59 full-text articles assessed were based on RCTs. However, 10 studies were excluded due to non-conforming interventions. Twenty-one studies involved participants diagnosed with *B. pertussis* infection and were excluded. In five studies, it was unclear whether only patients with PLS were included. Ultimately, 23 RCTs [18,19,20,21,22,23,24,25,26,27,28,29,30,31,32,33,34,35,36,37,38,39,40] were selected (Figure 1).

### 3.2. The Characteristics of the Study

All included 23 RCTs were conducted in China (although we did not apply any national restrictions) and published from 2015 to 2024. Sample sizes ranged from 40 to 140 participants, and the total treatment period ranged from 3 days to 2 weeks. The mean age distribution ranged from 9.89 ± 1.53 months to 6.26 ± 1.33 years. The duration of disease ranged from 5.2 ± 1.7 days to 35.71 ± 6.03 days. Most studies maintained identical total treatment durations for both the experimental and control groups (Table 1).

### 3.3. Interventions

Oral HMs were reported in all 23 studies. Twelve types of HM formulations were used, including Sangbaipi (SB) [18,19,20,21,25,34], Jiawei Weijing (WJ) [24,32,38], Chunggan Sapye decoction [28], Sosiho decoction with Sabaek powder [22], Canyu Dunke [23], Zhenhai Jingyan [27], Zhenhai Hwadam prescription [29], Dengtai Ye (DY) [30,35,36,37,40], Baikening [31], Xiaoer Feike granules [39], Lusika [26], and Heron Cough Pill [33]. The details of their compositions, dosages, and frequencies are provided in Table 2.

*Glycyrrhizae Radix et Rhizoma* was the most used ingredient, appearing in 16 RCTs, followed by *Armeniacae Semen* in 13 studies. *Mori Cortex Radicis* was included in 10 RCTs, whereas *Pinelliae Rhizoma* and *Perillae Fructus* were used in 9 RCTs each (Table S3).

In four studies [22,23,30,36], the experimental and control groups received additional treatments. All studies implemented respiratory support measures, such as inhalation therapy, nasal catheter use, and continuous positive airway pressure oxygen therapy. Three studies included antispasmodic treatments [23,30,36], whereas two studies aimed to relieve cough symptoms [30,36] (Table S4).

Western medications were also used for PLS treatment, including erythromycin [19,20,21,22,24,25,26,27,29,30,32,34,35,36,37,38,39,40], azithromycin [18,28], roxithromycin [33], ipratropium bromide [26], albuterol sulfate solution [26], budesonide [26,31], and montelukast [37].

### 3.4. Outcome Measures

Main outcome measures included TER [18,19,20,21,22,24,25,26,27,28,29,30,31,32,33,34,35,36,37,38,39,40], the disappearance time of the main symptoms (mainly spastic cough) [22,23,29,30,32,35,36,37,40], the recovery time of routine blood parameters to normal range [18,19,21,25,34,40], hospitalization time [18,19,20,21,22,25,29,34,36], and adverse events [24,27,28,30,31,35,36,37,38,40].

TER was reported in 22 studies [18,19,20,21,22,24,25,26,27,28,29,30,31,32,33,34,35,36,37,38,39,40] and demonstrated the objective effects of HM compared to WM alone. The disappearance time of the main symptoms, especially spastic cough, was an important evaluation indicator since spastic cough is a key symptom in diagnosing PLS. Although 15 studies [18,19,20,22,23,25,28,29,30,32,34,35,36,37,40] reported disappearance times for the main symptoms, only 9 studies [22,23,29,30,32,35,36,37,40] specified the exact symptoms analyzed.

The recovery time of routine blood parameters to normal range was included as an evaluation criterion. While many studies reported inflammatory factors in blood routine tests, their variability prevented meaningful analysis. Therefore, only studies assessing recovery to normal levels were included. Six studies [18,19,21,25,34,40] met this criterion, whereas eight studies [22,26,28,29,30,32,33,35] were excluded.

Hospitalization time was considered a relevant evaluation indicator, as effective treatments could shorten hospital stays. Nine studies [18,19,20,21,22,25,29,34,36] reported hospitalization time and were included in the analysis.

Adverse events were reported in 10 studies [24,26,27,30,31,35,36,37,38,40]. One study [26] was excluded because it only reported symptoms of adverse events rather than the number of occurrences. Further details on adverse events are presented in Table S5.

### 3.5. Meta-Analysis

The meta-analysis was performed to compare the outcomes with HM and WM combined versus WM alone. When significant heterogeneity was observed, subgroup analysis about the type of HM was conducted for further evaluation of outcomes.

#### 3.5.1. TER

**Figure 2:**
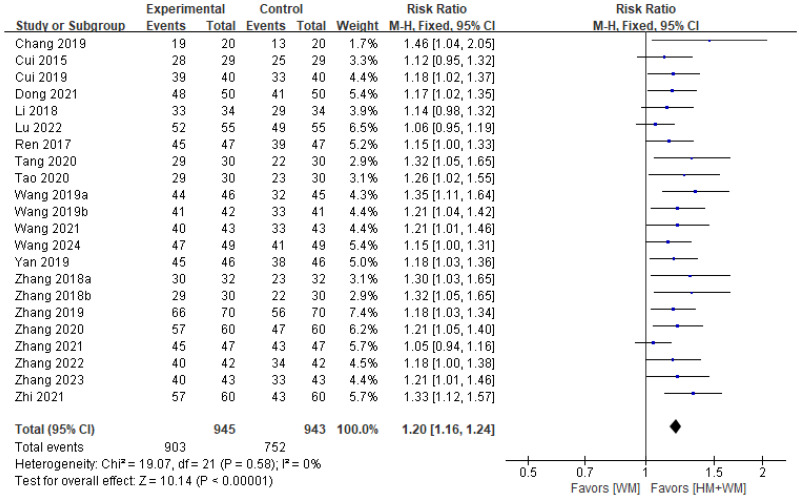
A forest plot of the total effective rate [18,19,20,21,22,24,25,26,27,28,29,30,31,32,33,34,35,36,37,38,39,40].

A total of 22 studies [18,19,20,21,22,24,25,26,27,28,29,30,31,32,33,34,35,36,37,38,39,40], involving 1888 patients, reported TER and were included in the analysis. The results indicated no significant heterogeneity (*p* = 0.58, I^2^ = 0%), and a fixed-effects model was used. The analysis demonstrated that HM with WM was more effective than WM in improving TER (RR = 1.20; 95% CI: 1.16–1.24; *p* < 0.001, Figure 2). Since there was no heterogeneity and an adequate number of RCTs were included, additional subgroup analysis was not required.

#### 3.5.2. Disappearance Time of Main Symptoms (Spastic Cough)

**Figure 3:**
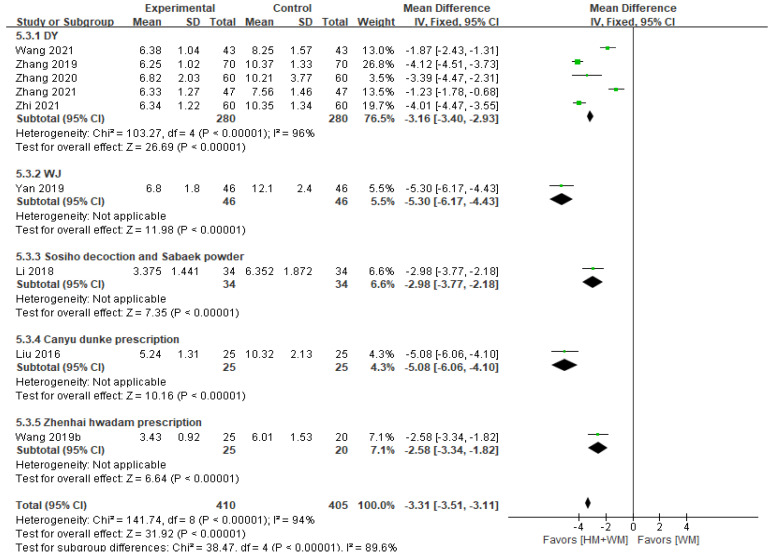
A forest plot of the disappearance time of main symptoms (spastic cough). DY, Dengtai ye granule; WJ, Jiawei Weijing decoction [22,23,29,30,32,35,36,37,40].

Nine studies [22,23,29,30,32,35,36,37,40], including 815 patients, reported the disappearance time of spastic cough. The meta-analysis showed that HM significantly reduced the disappearance time of spastic cough compared to WM alone (fixed-effects MD = −3.31, 95% CI: −3.51–−3.11, Figure 3). However, substantial heterogeneity was observed (*p* < 0.001, I^2^ = 94%). Subgroup analysis indicated that WJ was the most effective in reducing disappearance time (MD = −5.30, 95% CI: −6.17–−4.43).

#### 3.5.3. Recovery Time of Blood Routine Parameters to Normal Range

**Figure 4:**
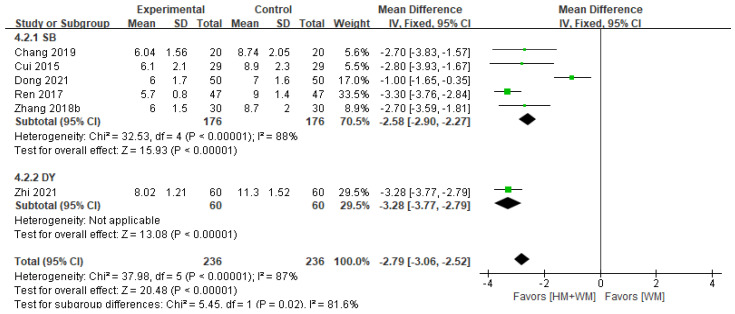
A forest plot of the **r**ecovery time of blood routine to normal range. DY, Dengtai ye granule; SB, Sangbaipi decoction [18,19,21,25,34,40].

Six studies [18,19,21,25,34,40], involving 472 patients, assessed the recovery time of routine blood parameters to normal levels. HM was more effective than WM alone in accelerating recovery (fixed-effects MD = −2.79, 95% CI: −3.06–−2.52, I^2^ = 87%, *p* < 0.001, Figure 4). Subgroup analysis identified SB (MD = −2.58, 95% CI: −2.90–−2.27) and DY (MD = −3.25, 95% CI: −3.77–−2.79) as effective interventions for PLS.

#### 3.5.4. Hospitalization Time

**Figure 5:**
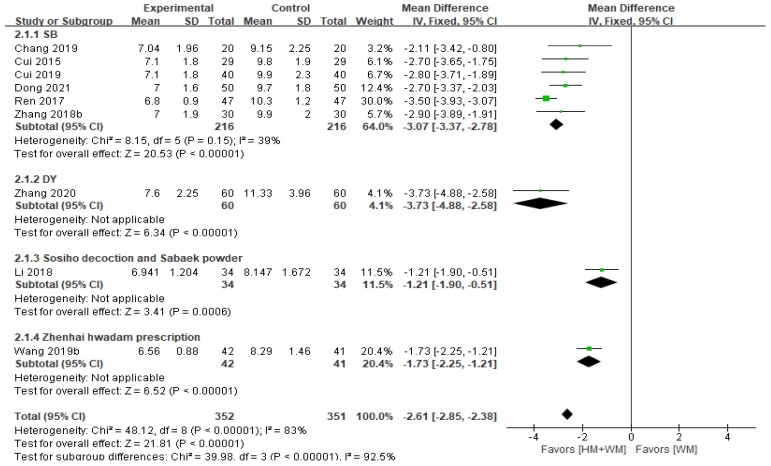
A forest plot of the hospitalization time. DY, Dengtai ye granule; SB, Sangbaipi decoction [18,19,20,21,22,25,29,34,36].

Nine studies [18,19,20,21,22,25,29,34,36], with 703 patients, assessed hospitalization time. The analysis revealed that HM with WM significantly shortened hospitalization time (MD = −2.61, 95% CI: −2.85–−2.38, Figure 5). Subgroup analysis showed that SB (MD = −3.07, 95% CI: −3.37–−2.78) and DY (MD = −3.73, 95% CI: −4.88–−2.58) were more effective in reducing hospitalization time than other herbal formulations.

### 3.6. Adverse Events

Nine studies [24,27,30,31,35,36,37,38,40] reported adverse events, involving 912 patients (456 each in the HM and WM groups). Only one study [35] reported no adverse events. In the HM with WM group, there were 27 cases of adverse events, whereas the WM group had 40 cases. The most frequently reported adverse events in the HM group included nausea and vomiting (nine cases), diarrhea, abdominal distension, abdominal pain, and increased frequency of defecation (each four cases). The WM group also reported nausea and vomiting (ten cases), diarrhea (seven cases), abdominal pain (five cases), abdominal distension (four cases), increased frequency of defecation, and abdominal discomfort (three cases). Other symptoms, such as rash and constipation, were rarely reported in either group. All adverse events were mild and resolved without additional treatments.

### 3.7. Assessment of Reporting Bias

**Figure 6:**
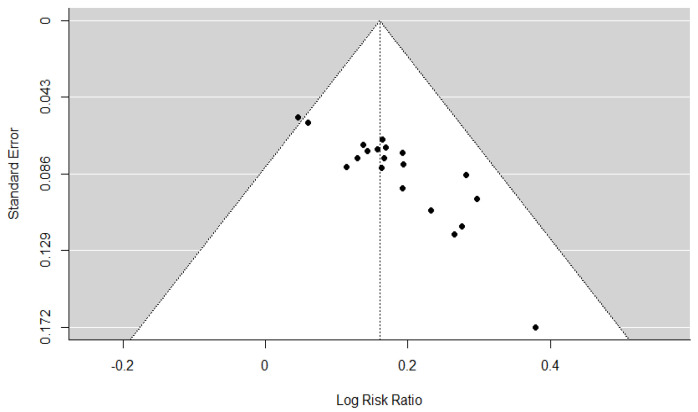
Funnel plot of the total effective rate.

**Figure 7:**
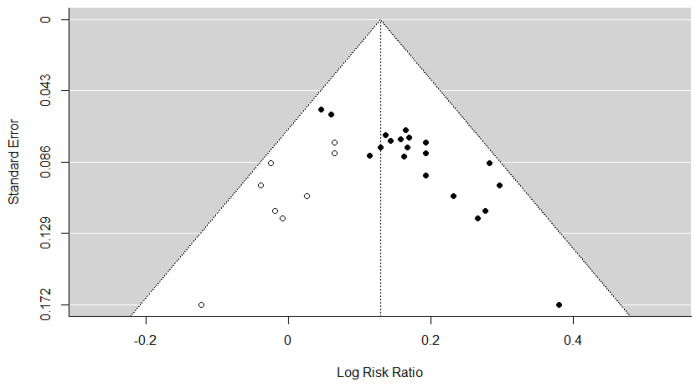
Adjusted funnel plot (trim and fill) of the total effective rate. The closed dots represent real studies and the open dots represent filled artificial studies.

Publication bias for the TER was assessed. Publication bias was not assessed for other outcome measures owing to the inclusion of <10 studies. The assessment of publication bias revealed significant funnel plot asymmetry based on Egger’s regression test (t = 7.4624, *p* < 0.001; Figure 6), indicating the presence of potential small-study effects. The trim-and-fill method estimated that nine studies might be missing from the left side of the funnel plot (Figure 7), suggesting a possible overestimation of the intervention effect. After adjusting for these potentially missing studies, the pooled effect size was logRR = 0.1287 (95% CI: 0.0989–0.1584, *p* < 0.001), corresponding to an RR of 1.14 (95% CI: 1.10–1.17). Although slightly attenuated compared with the original estimate of RR = 1.17 (logRR = 0.1603), the result remained statistically significant. Furthermore, the Rosenthal fail-safe N was 786, far exceeding the commonly recommended minimum threshold of 120 (calculated as 5k + 10, where k = 22 is the number of included RCTs), indicating that 786 unpublished null-result studies would be required to negate the observed results. These findings suggest potential publication bias. However, the robustness of the conclusions was maintained.

### 3.8. Subgroup and Sensitivity Analyses

Subgroup analysis based on specific HM formulations was performed in the meta-analysis. For TER, no additional subgroup analysis was conducted owing to the sufficient number of RCTs. Sensitivity analyses were not performed independently for other outcomes because <10 RCTs were included in each category (Table S6).

### 3.9. Quality Assessment

Except for one study [22], concerns regarding bias were raised in the randomization process of the included RCTs. Allocation sequence concealment was not reported in any RCTs, but no baseline imbalances were observed. However, in one study [22], insufficient information regarding the randomization process was reported.

All included studies had some concerns regarding deviations from intended interventions as a result of insufficient relevant information. Most studies were evaluated to have a low risk of bias in outcome measurements, with the exception of one study [23], where differences between the stated methodology and reported results led to a high risk.

Outcome measurements were evaluated using appropriate indicators, leading to a low risk in this category. In terms of the selection of reported results, all studies were rated as having a low risk.

**Figure 8:**
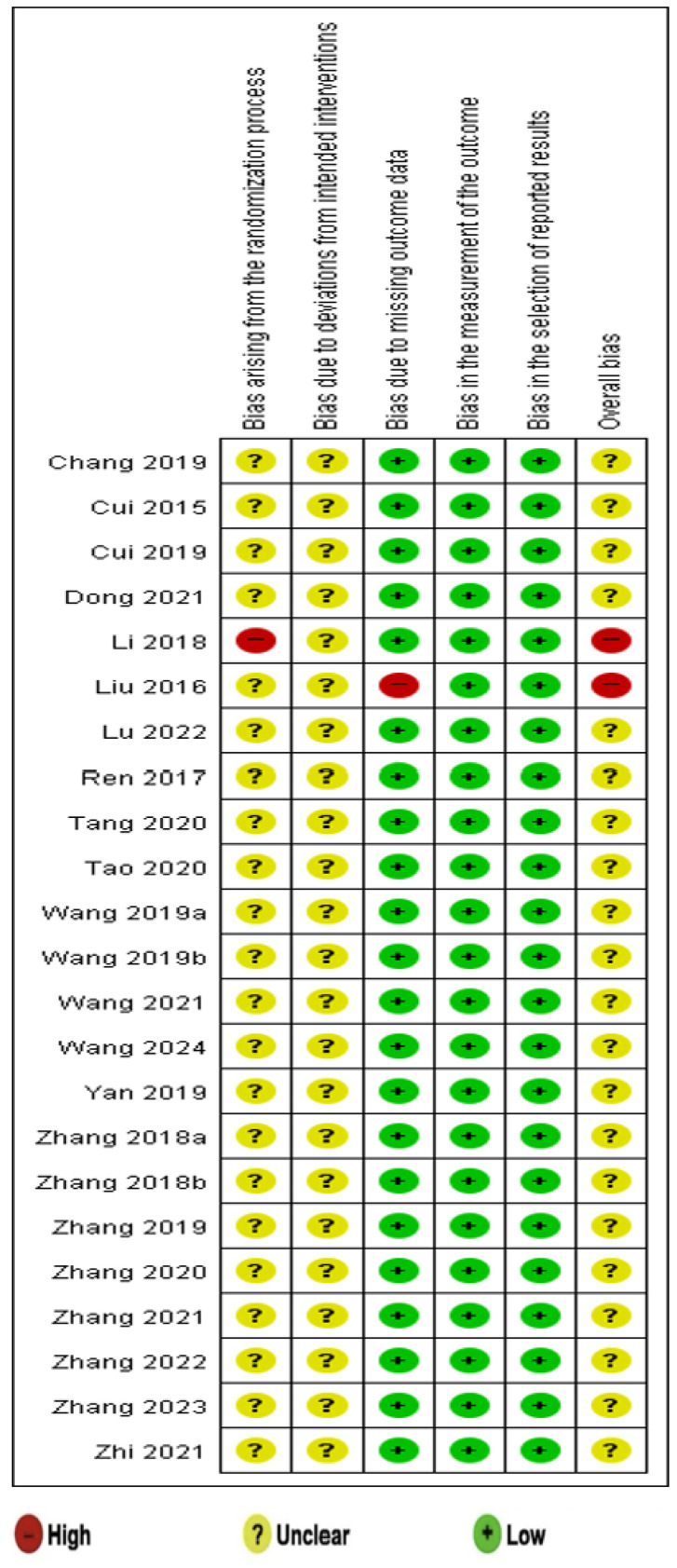
Risk of bias assessment [18,19,20,21,22,24,25,26,27,28,29,30,31,32,33,34,35,36,37,38,39,40].

**Figure 9:**
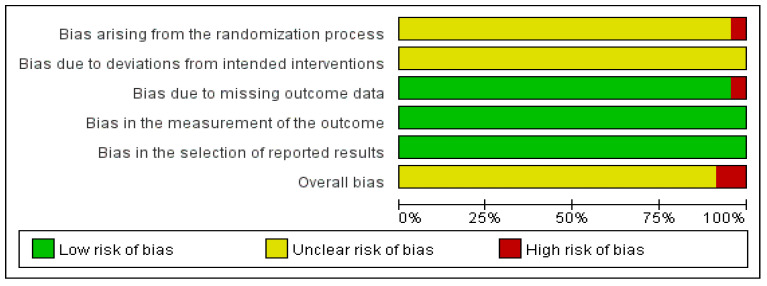
Risk of bias graph.

With the exception of two studies [22,23] categorized as “high risk”, all other RCTs were rated as having “some concerns (unclear)” regarding bias. These findings are visualized in Figure 8 and Figure 9.

### 3.10. GRADE Certainty of Evidence

The GRADE evidence profile is presented in Table 3. The TER (overall), disappearance time of main symptoms (spastic cough) (overall and DY subgroup), recovery time of routine blood parameters to normal range (overall), and hospitalization time (overall and SB subgroup) were rated as “moderate quality”. Owing to a high risk of bias and imprecision, other outcomes were rated as having a “low” or “very low” quality.

## 4. Discussion

### 4.1. Summary of Findings

The findings of this study indicate that combining HM with WM provides greater therapeutic benefits than WM alone in pediatric PLS. Data from 23 RCTs conducted in China involving 1938 children were analyzed. Children with PLS in these RCTs were divided into the control group if they used WM and the experimental group if they used a combination of HM and WM, and they received the corresponding treatments for less than two weeks. However, a high heterogeneity for all outcomes, except for TER, was observed among the studies because they covered 12 different types of HMs. Accordingly, subgroup analyses were conducted based on specific HM types.

A meta-analysis revealed that HM significantly improved the TER and other clinical outcomes. In the included RCTs, the TER was assessed by reflecting the degree of improvement in spastic cough and the main symptoms of PLS, such as lung rhonchus sounds, wheezing and wet chirping, panting, and shortness of breath. This observation highlights the appropriateness of using TER as the main outcome measure for PLS, where the diagnosis was based on symptoms. These findings suggest that HM in combination with WM can help alleviate PLS symptoms. In particular, JW was effective in reducing the disappearance time of main symptoms, especially spastic cough. Additionally, HM accelerated the recovery of blood routine parameters, with DY showing the greatest efficacy, followed by SB. DY and SB were also more effective in shortening hospitalization time than WM alone.

Across all included studies, digestive symptoms such as nausea (or vomiting), abdominal pain, and diarrhea were the most reported adverse events. However, their occurrence was lower in patients who were treated with HM combined with WM than in patients treated with WM alone. All reported adverse events were mild and resolved without the need for additional treatment.

Regarding risk of bias, most RCTs had “some concerns”, and two RCTs were rated as having a “high” risk of bias. The main issues involved randomization procedures and deviations from intended interventions, with only one RCT having missing outcome data. Due to the lack of number of RCTs per outcome, publication bias could only be evaluated for TER.

This study is significant as it addresses the use of HM in treating PLS, a condition that has symptoms similar to pertussis in children and is often overlooked in clinical practice.

### 4.2. Clinical Implications

PLS closely resembles whooping cough, presenting with paroxysmal (spastic) cough, facial redness, and often a high-pitched inspiratory “whoop” sound [41]. As with whooping cough, PLS can manifest a variety of respiratory symptoms, with persistent spastic cough being a key diagnostic feature. In severe cases, prolonged coughing may lead to complications, such as vomiting, dehydration, apnea, cyanosis, convulsions, and death in young children [42,43]. Several studies have identified pathogens, such as adenovirus, *Mycoplasma pneumoniae*, and influenza B virus, as potential causes of PLS [44,45]. However, due to the wide range of possible causative pathogens, treatment generally focuses on symptomatic management rather than pathogen-specific therapy.

Macrolide antibiotics, including erythromycin and azithromycin, are commonly used to treat respiratory infections and are standard treatments for both whooping cough and PLS [46]. However, while antibiotics effectively eliminate pathogens, they do not significantly alleviate persistent clinical symptoms [12]. Moreover, these medications are associated with digestive adverse events, such as nausea (or vomiting), abdominal pain, and diarrhea, with erythromycin having a particularly high likelihood of causing such adverse effects [47]. Azithromycin and roxithromycin are next-generation antibiotics that cause fewer adverse digestive effects than those caused by erythromycin. They are prescribed to children; however, caution is warranted when prescribing these drugs at a high dosage [48,49]. Therefore, it is important to explore whether HM can improve symptoms without exacerbating the gastrointestinal adverse effects when used alongside WM, such as with antibiotics. In addition to antibiotics, WMs generally used to treat respiratory failure in children, such as asthma, were used to treat PLS symptoms in the included RCTs [50]. Ipratropium bromide (an anticholinergic drug), albuterol sulfate (a beta agonist), and montelukast (a leukotriene receptor antagonist) are bronchodilators that improve lung function, control symptoms, and reduce exacerbations of asthma [51]. Budesonide (a corticosteroid) acts as an anti-inflammatory agent and is often used in combination with bronchodilators to achieve maximum efficacy [52]. However, anticholinergic drugs and beta-agonists can cause increased heart rate, tremors, and palpitations [53]. Additionally, whether montelukast causes neuropsychiatric adverse events in children remains debatable [54]. In particular, inhaled corticosteroids are used for long-term symptoms, and there are concerns that they affect children’s growth, adrenal function, and bone density [55]. In this study, it was expected that HMs could play the role of WMs in alleviating symptoms.

This systematic review analyzed 12 types of oral HMs used to treat PLS. Among them, SB, WJ, and DY were evaluated in multiple studies. SB is traditionally administered for respiratory disorders induced by phlegm–heat obstruction and has demonstrated anti-influenza and anti-inflammatory effects [56]. *Mori Cortex Radicis*, a key ingredient of SB, has strong anti-inflammatory properties [57]. Additionally, it elevates the expression of regulatory T cells and represses Th2 cytokines in asthma and acts as an anti-allergic HM similar to the WMs used in the included RCTs [58]. Similarly, WJ is used to clear phlegm and heat from the lungs, improve lung function, and regulate arterial blood gases. It reportedly reduces the expression of inflammatory markers in respiratory disorders, such as chronic obstructive pulmonary disease (COPD) [59]. Its active compounds—beta-sitosterol, tricin, and stigmasterol—are reportedly beneficial for conditions such as pneumonia and COPD [60]. *Folium Wrightiae Laevis*, derived from the leaves of *Alstonia scholaris* (L.) *R. Br.*, is traditionally used for treating lung fever, cough, and phlegm, and DY has been developed for managing respiratory disorders such as chronic bronchitis [61].

*Glycyrrhizae Radix et Rhizoma*, the most frequent ingredient in included RCTs (present in 16 prescriptions), has anti-inflammatory and antiviral properties and can enhance the efficacy of other herbs [62,63,64]. Particularly, triterpene saponin, the main chemical component, exhibits antimicrobial and antiviral activities against infectious respiratory diseases such as coronavirus, influenza, and COVID-19 [65]. The second most used herb, *Armeniacae Semen*, has been widely recognized for its benefits in treating lung diseases such as cough and asthma [66]. Notably, its active component, amygdalin, exhibits strong anti-inflammatory and antifibrotic properties [67]. Amygdalin also inhibits cell death and prevents cell damage in lung epithelial cells infected with bacteria [68]. *Pinelliae Rhizoma* is also a notable herb, and its components, chrysin, DHC, and 7,8-DHF, control the inflammatory response by suppressing the activity of the NLRP3 inflammasome due to acute infection of the lungs [69]. This study found that the HMs (herbs) used in RCTs could play a role similar to that of conventional WMs, without increasing gastrointestinal adverse events.

To support the effects of the above-mentioned HMs, this study analyzed whether the herbs were effective in improving major symptoms of PLS, such as spastic cough, reducing the time for routine blood parameters to recover to normal range, and shortening hospitalization time. The findings suggest that combining HM with WM is more effective in treating PLS symptoms in children than WM alone.

### 4.3. Limitations and Suggestions

This study has several limitations. First, although databases in four languages were searched without regional restrictions, all the included RCTs were performed in China. However, considering that HMs are widely used in East Asia, particularly China, this geographical concentration alone should not be interpreted as indicative of low methodological quality or substantial heterogeneity among studies. Additionally, if future studies included RCTs from other regions, they could provide a more comprehensive evaluation of the efficacy of HMs.

Second, most RCTs have challenges in blinding participants, leading to a potential risk of bias. Additionally, heterogeneity was observed, and the evidence quality ranged from moderate to very low, suggesting the need for well-designed studies with standardized methodologies.

Third, this study assessed publication bias only with respect to the TER. The funnel plot of TER revealed asymmetry, suggesting potential publication bias. Additional analysis revealed that these results did not affect the overall conclusion. However, it was difficult to assess publication bias for other outcomes that were based on less than 10 RCTs because of insufficient data and diversity among HMs used. These factors contributed to the high heterogeneity in the results of other outcomes. When designing future RCTs on PLS, it is necessary to select consistent outcome indicators for comparison. Additionally, if sufficient RCTs are available, a meta-analysis can be conducted to determine the effectiveness of a specific type of HM in pediatric patients with PLS.

Fourth, inflammatory markers, such as C-reactive protein, interleukins, and immunoglobulins, are important indicators of treatment efficacy. However, non-uniform measurement methods across studies prevented any direct comparisons. Future studies incorporating these biomarkers would provide more objective and reliable evidence.

Finally, only one included study [27] reported the recurrence rate of PLS. Because PLS can recur due to various pathogens, future studies should use recurrence rates as an indicator of the long-term efficacy of HM in managing PLS.

In conclusion, this study statistically showed that a combination of HM and WM is more effective than treatment with WM alone in children with PLS. However, high-quality, well-designed RCTs of PLS need to be conducted globally.

## 5. Conclusions

This study showed that HM combined with WM is more effective than WM alone for managing PLS in children, and the combination does not lead to serious adverse events. However, the quality of evidence was not high, highlighting the need for well-designed, high-quality RCTs in the future. Additionally, owing to the inconsistency in the types of HMs used, further studies focusing on specific HM formulations are necessary. Despite these limitations, our findings provide valuable insights for clinicians, particularly when managing PLS in children who do not meet the diagnostic criteria for whooping cough.

## Figures and Tables

**Table 1:** Basic characteristics of the included studies.

First Author (Year)	Study Location	Sample Size (E/C)	Gender (M/F)	Age Distribution (Mean ± SD)	Duration of Disease (Mean ± SD)	Experimental Intervention (E)	Total Treatment Periods	Outcome Measurement
						Control Intervention (C)		
Chang (2019) [18]	China	40 (20/20)	E: 20 (12/8) C: 20 (11/9)	E: (3.42 ± 0.53) yr C: (3.35 ± 0.49) yr	E: (15.24 ± 2.61) d C: (15.18 ± 2.56) d	(C) + HM	10–12 d	(1)(2)(3)(4)(5) (6)
						Azithromycin		
Cui (2015) [19]	China	58 (29/29)	E: 29 (16/13) C: 29 (15/14)	E: (2.3 ± 1.8) yr C: (2.6 ± 1.8) yr	(8.75± 2.1) d	(C) + HM	2 wk	(1)(4)(5)(6)
						Erythromycin		
Cui (2019) [20]	China	80 (40/40)	E: 40 (23/17) C: 40 (21/19)	E: (3.5 ± 0.6) yr C: (3.7 ± 0.3) yr	E: (15.2 ± 2.6) d C: (15.6 ± 2.5) d	(C) + HM	2 wk	(1)(2)(4)(6)
						Erythromycin		
Dong (2021) [21]	China	100 (50/50)	E: 50 (28/22) C: 50 (30/20)	E: (3.0 ± 1.8) yr C: (2.9 ± 1.66) yr	NR	(C) + HM	NR	(1)(5)(6)
						Erythromycin		
Li (2018) [22]	China	68 (34/34)	NR	NR	NR	(C) + HM	NR	(1)(4)(6)
						Erythromycin + CT		
Liu (2016) [23]	China	50 (25/25)	E: 25 (15/10) C: 25 (13/12)	E: (5.32 ± 1.68) yr C: (5.78 ± 1.21) yr	E: (10.8± 4.6) d C: (11.3± 5.3) d	(C) + HM	2 wk	(2)(4)(7)
						CT		
Lu (2022) [24]	China	110 (55/55)	E: 55 (28/27) C: 55 (30/25)	E: (2.46 ± 0.71) yr C: (2.23 ± 0.65) yr	E: (14.27 ± 1.21) d C: (14.23 ± 1.13) d	(C) + HM	2 wk	(1)(2)(3)(8)
						Erythromycin + Sodium Ascorbate		
Ren (2017) [25]	China	94 (47/47)	E: 47 (27/20) C: 47 (28/19)	E: (2.5 ± 1.2) yr C: (2.6 ± 1.3) yr	E: (8.9 ± 1.8) d C: (8.8 ± 1.9) d	(C) + HM	3 d	(1)(4)(5)(6)(8)
						Erythromycin		
Tang (2020) [26]	China	60 (30/30)	E: 30 (16/14) C: 30 (17/13)	E: (2.4 ± 0.8) yr C: (2.5 ± 0.7) yr	E: (5.2 ± 1.7) d C: (5.3 ± 1.8) d	(C) + HM	2 wk	(1)
						Erythromycin + Budesonide, Ipratropium Bromide, Albuterol Sulfate Solution		
Tao (2020) [27]	China	60 (30/30)	E: 30 (18/12) C: 30 (19/11)	NR	NR	(C) + HM	2 wk	(1)(2)(8)(9)
						Azithromycin		
Wang (2019a) [28]	China	91 (46/45)	E: 46 (25/21) C: 45 (23/22)	E: (1.55 ± 0.58) yr C: (1.63 ± 0.54) yr	E: (19.28 ± 2.79) d C: (20.16 ± 2.33) d	(C) + HM	2 wk	(1)(4)
						Erythromycin		
Wang (2019b) [29]	China	83 (41/42)	E: 41 (22/19) C: 42 (21/21)	E: (10.01 ± 1.39) m C: (9.89 ± 1.53) m	E: (33.69 ± 5.13) d C: (35.01 ± 6.03) d	(C) + HM	2 wk	(1)(4)(6)(8)
						Erythromycin		
Wang (2021) [30]	China	86 (43/43)	E: 43 (28/15) C: 43 (26/17)	E: (1.55 ± 0.58) yr C: (1.63 ± 0.54) yr	E: (35.69 ± 1.25) d C: (35.71 ± 6.03) d	(C) + HM	2 wk	(1)(4)
						Erythromycin + CT		
Wang (2024) [31]	China	98 (49/49)	E: 49 (28/15) C: 49 (26/17)	E: (3.14 ± 0.89) yr C: (3.22 ± 0.81) yr	E: (10.36 ± 3.18) d C: (10.18 ± 2.42) d	(C) + HM	10 d	(1)(2)(3)
						Budesonide		
Yan (2019) [32]	China	92 (46/46)	E: 46 (30/16) C: 46 (29/17)	E: (2.47 ± 0.53) yr C: (2.56 ± 0.46) yr	E: (12.50 ± 2.81) d C: (12.91 ± 2.30) d	(C) + HM	2 wk	(1)(4)
						Erythromycin		
Zhang (2018a) [33]	China	64 (32/32)	E: 32 (18/14) C: 32 (19/13)	NR	NR	(C) + HM	10 d	(1)
						Roxithromycin		
Zhang (2018b) [34]	China	60 (30/30)	E: 30 (15/15) C: 30 (16/14)	E: (3.4 ± 0.8) yr C: (3.6 ± 0.7) yr	E: (15.5 ± 2.5) d C: (15.0 ± 2.7) d	(C) + HM	2 wk	(1)(4)(5)(6)
						Erythromycin		
Zhang (2019) [35]	China	140 (70/70)	E: 70 (39/31) C: 70 (37/33)	E: (5.62 ± 1.30) yr C: (5.59 ± 1.29) yr	NR	(C) + HM	2 wk	(1)(4)
						Erythromycin		
Zhang (2020) [36]	China	120 (60/60)	E: 60 (35/25) C: 60 (32/28)	E: (6.26 ± 1.33) yr C: (6.09 ± 1.42) yr	E: (14.31 ± 3.42) d C: (14.70 ± 3.05) d	(C) + HM	2 wk	(1)(2)(4)(6)(10)
						Erythromycin + CT		
Zhang (2021) [37]	China	94 (47/47)	E: 47 (21/26) C: 47 (23/24)	E: (3.33 ± 1.39) yr C: (3.28 ± 1.42) yr	E: (13.24 ± 1.91) d C: (13.45 ± 1.86) d	(C) + HM	2 wk	(1)(4)(10)
						Erythromycin + Feilike HeJi, Montelukast		
Zhang (2022) [38]	China	84 (42/42)	E: 42 (25/17) C: 42 (23/19)	E: (4.06 ± 1.32) yr C: (4.37 ± 1.27) yr	E: (13.19 ± 2.91) d C: (13.52 ± 2.86) d	(C) + HM	2 wk	(1)(2)(3)(8)
						Erythromycin		
Zhang (2023) [39]	China	86 (43/43)	E: 43 (22/21) C: 43 (23/20)	E: (1.47 ± 0.33) yr C: (1.43 ± 0.35)yr	E: (15.48 ± 3.22) d C: (15.84 ± 3.38) d	(C) + HM	2 wk	(1)(3)(10)
						Erythromycin		
Zhi (2021) [40]	China	120 (60/60)	E: 60 (31/29) C: 60 (30/30)	E: (5.62 ± 1.32) yr C: (5.73 ± 1.30) yr	E: (10 ± 3.0) d C: (10 ± 3.2) d	(C) + HM	2 wk	(1)(2)(5)
						Erythromycin		

E, experimental; C, control; SD, standard deviation; NR, not reported; d, day; wk, week; yr, year; HM, herbal medicine; CT, conventional treatment; (1), total effective rate; (2), inflammatory factor level; (3), pulmonary function index; (4), disappearance time of main symptoms and pulmonary signs (spastic cough, lung rhonchus sound, wheezing and wet chirping, panting, shortness of breath); (5), recovery time of blood routine to normal range; (6), hospitalization time; (7), number of sputum suctions; (8), symptom scores; (9), recurrence rate; (10), cellular immune function.

**Table 2:** Herbal medicine information.

First Author (Year)	Prescription /Composition	Dosage (Day)	Frequency (Day)
Chang (2019) [18]	Sangbaipi decoction *Mori Cortex Radicis* 10 g, *Pinelliae Rhizoma* 10 g, *Armeniacae Semen* 10 g, *Lepidii seu Descurainiae Semen* 10 g, *Stemonae Radix* 10 g, *Perillae Fructus* 6 g, *Scutellariae Radix* 6 g, *Glycyrrhizae Radix et Rhizoma* 6 g, *Ilicis pubescentis Radix* 15 g, *Meretricis Concha* 15 g, *Ficus hirta vahl.* 15 g, *Tribuli Fructus* 5 g, *Paridis Rhizoma* 8 g, *Aristolochiae Fructus* 3 g	0–1 yr: 30–100 mL 1–6 yr: 100–200 mL	2 times
Cui (2015) [19]	Sangbaipi decoction *Mori Cortex Radicis* 10 g, *Pinelliae Rhizoma* 10 g, *Armeniacae Semen* 10 g, *Fritillariae Thunbergii Bulbus* 10 g, *Perillae Fructus* 10 g, *Persicae Semen* 10 g, *Lepidii seu Descurainiae Semen* 10 g, *Scutellariae Radix* 6 g, *Glycyrrhizae Radix et Rhizoma* 6 g	<1 yr: 30–100 mL 1–6 yr: 100–200 mL	1 time
Cui (2019) [20]	Sangbaipi decoction *Scutellariae Radix* 6 g, *Persicae Semen* 10 g, *Perillae Fructus* 10 g, *Mori Cortex Radicis* 10 g, *Lepidii seu Descurainiae Semen* 10 g, *Fritillariae Thunbergii Bulbus* 10 g, *Pinelliae Rhizoma* 10 g, *Armeniacae Semen* 10 g, *Glycyrrhizae Radix et Rhizoma* 6 g	200 mL	1 time
Dong (2021) [21]	Sangbaipi decoction *Persicae Semen* 10 g, *Mori Cortex Radicis* 10 g, *Fritillariae Thunbergii Bulbus* 10 g, *Armeniacae Semen* 10 g, *Pinelliae Rhizoma* 10 g, *Lepidii seu Descurainiae Semen* 10 g, *Perillae Fructus* 10 g, *Glycyrrhizae Radix et Rhizoma* 6 g, *Scutellariae Radix* 6 g	<1 yr: 30–100 mL 1–6 yr: 100–200 mL	1 time
Li (2018) [22]	Sosiho decoction and Sabaek powder *Bupleuri Radix* 9 g, *Scutellariae Radix* 6 g, *Pinelliae Rhizoma* 9 g, *Zingiberis Rhizoma Recens* 3 g, *Mori Cortex Radicis* 10 g, *Lycii Radicis Cortex* 10 g, *Armeniacae Semen* 10 g, *Fritillariae Cirrhosae Bulbus* 10 g, *Lepidii seu Descurainiae Semen* 10 g, *Batryticatus Bombyx* 10 g, *Lumbricus* 10 g, *Cicadidae Periostracum* 10 g, *Glycyrrhizae Radix et Rhizoma* 6 g	NR	NR
Liu (2016) [23]	Canyu dunke prescription *Schizonepetae Spica* 3 g, *Saposhnikoviae Radix* 3 g, *Platycodonis Radix* 6 g, *Armeniacae Semen* 5 g, *Asteris Radix et Rhizoma* 6 g, *Stemonae Radix* 5 g, *Poria* 3 g, *Farfarae Flos* 6 g, *Lonicerae Flos* 6 g, *Fritillariae Cirrhosae Bulbus* 3 g, *Evodiae Fructus* 3 g, *Pinelliae Rhizoma* 3 g, *Codonopsis Pilosulae Radix* 5 g, *Glycyrrhizae Radix et Rhizoma* 3 g	NR	NR
Lu (2022) [24]	Jiawei weijing decoction *Phragmitis Rhizoma* 15 g, *Persicae Semen* 10 g, *Coicis Semen* 5 g, *Benincasae Semen* 5 g, *Lonicerae Flos* 5 g, *Forsythiae Fructus* 5 g, *Glycyrrhizae Radix et Rhizoma* 6 g	NR	>4 yr: 2 times <4 yr: NR
Ren (2017) [25]	Sangbaipi decoction *Glycyrrhizae Radix et Rhizoma* 6 g, *Scutellariae Radix* 6 g, *Mori Cortex Radicis* 10 g, *Lepidii seu Descurainiae Semen* 10 g, *Pinelliae Rhizoma* 10 g, *Perillae Fructus* 10 g, *Fritillariae Thunbergii Bulbus* 10 g, *Armeniacae Semen* 10 g, *Persicae Semen* 10 g	<1 yr: 30–100 mL 1–6 yr: 100–200 mL	2 times
Tang (2020) [26]	Lusika pill * (Beijing Tongrentang Co., Ltd. (Beijing, China) Tongrentang Pharmaceutical Factory) *Armeniacae Semen, Gypsum Fibrosum*, *Glycyrrhizae Radix et Rhizoma*, *Asiasari Radix et Rhizoma*, *Perillae Fructus*, *Brassicae Semen*, *Arctii Fructus*, *Trichosanthis Pericarpium*, *Belamcandae Rhizoma*, *Indigo Pulverata Levis*, *Meretricis Concha*, *Trichosanthis Radix*, *Gardeniae Fructus*, *Artificial Bovis Calculus*, *Ephedrae Herba*, *Glycyrrhizae Radix et Rhizoma*	3 g	2 times
Tao (2020) [27]	Zhenhai jingyan prescription (New Green Pharmaceutical Co., Ltd. (Sichuan, China)) *Mori Cortex Radicis* 8 g, *Lycii Radicis Cortex* 8 g, *Aurantii Fructus Immaturus* 6 g, *Armeniacae Semen* 8 g, *Eriobotryae Folium* 10 g, *Stemonae Radix* 10 g, *Scorpio* 1 g *Fritillariae Thunbergii Bulbus* 8 g, *Belamcandae Rhizoma* 10 g, *Lumbricus* 6 g, *Arisaematis Rhizoma* 3 g,	60–100 mL	2 times
Wang (2019a) [28]	Chunggan sapye decoction *Bupleuri Radix* 10 g, *Cicadidae Periostracum* 6 g, *Lumbricus* 3 g, *Scutellariae Radix* 6 g, *Pinelliae Rhizoma* 6 g, *Armeniacae Semen* 9 g, *Fritillariae Thunbergii Bulbus* 6 g, *Lepidii seu Descurainiae Semen* 9 g, *Batryticatus Bombyx* 6 g, *Zingiberis Rhizoma Recens* 3 g, *Mori Cortex Radicis* 10 g, *Lycii Radicis Cortex* 10 g	0.5 pack	2 times
Wang (2019b) [29]	Zhenhai hwadam prescription *Batryticatus Bombyx* 9 g, *Gastrodiae Rhizoma* 9 g, *Aconiti Koreani Tuber* 6 g, *Aconiti Lateralis Radix Preparata* 3 g, *Aucklandiae Radix* 6 g, *Scorpio* 3 g, *Syzygii Flos* 3 g, *Citri Unshius Pericarpium* 6 g, *Pinelliae Tuber* 3 g, *Glycyrrhizae Radix et Rhizoma* 3 g	<2 m: 20 mL 2–5 m: 30 mL 6–8 m: 40 mL 9–12 m: 50 mL >12 m: 60 mL	2 times
Wang (2021) [30]	Dengtai ye granules * (Yunnan Botanical Pharmaceutical Co., Ltd. (Kunming, China), Z53020312) *Folium Wrightiae Laevis*	30 g	3 times
Wang (2024) [31]	Baikening granules * (Guangdong sencee pharmaceutical Co., Ltd. (Guangzhou, China), Z20050140) *Ginkgonis Semen*, *Indigo Pulverata Levis*, *Fritillariae Thunbergii Bulbus*	≤1 yr: 3 pack 1–3 yr: 4.5 pack ≥3 yr: 6 pack	3 times
Yan (2019) [32]	Jiawei weijing decoction *Phragmitis Rhizoma* 15 g, *Stemonae Radix* 10 g, *Paeoniae Radix* 10 g, *Persicae Semen* 10 g, *Coicis Semen* 6 g, *Benincasae Semen* 6 g, *Arisaematis Rhizoma* 3 g, *Armeniacae Semen* 6 g, *Imperatae Rhizoma* 6 g, *Perillae Fructus* 6 g, *Scorpio* 2 g, *Peucedani Radix* 6 g, *Plantaginis Semen* 6 g, *Glycyrrhizae Radix et Rhizoma* 3 g	<1 yr: 2/3 pack 1–3 yr: 1 pack 3–5 yr: 4/3 pack ≥5 yr: 2 pack	2 times
Zhang (2018a) [33]	Heron cough pill * Ephedrae Herba, *Asiasari Radix et Rhizoma*, *Arctii Fructus*, *Gypsum Fibrosum*, *Trichosanthis Radix*, *Gardeniae Fructus*, *Indigo Pulverata Levis*, *Glycyrrhizae Radix et Rhizoma*, *Armeniacae Semen*	6–12 m: 1.5 g 1–3 yr: 3.0 g	2 times
Zhang (2018b) [34]	Sangbaipi decoction *Persicae Semen* 10 g, *Mori Cortex Radicis* 10 g, *Fritillariae Thunbergii Bulbus* 10 g, *Pinelliae Rhizoma* 10 g, *Lepidii seu Descurainiae Semen* 10 g, *Perillae Fructus* 10 g, *Glycyrrhizae Radix et Rhizoma* 6 g, *Scutellariae Radix* 6 g	<1 yr: 30–100 mL 1–6 yr: 100–200 mL	1 time
Zhang (2019) [35]	Dengtai ye granules * (Yunnan Botanical Pharmaceutical Co., Ltd. (Kunming, China) Z53020312) *Folium Wrightiae Laevis*	30 g	3 times
Zhang (2020) [36]	Dengtai ye granules * (Yunnan Botanical Pharmaceutical Co., Ltd. (Kunming, China), Z53020312) *Folium Wrightiae Laevis*	30 g	3 times
Zhang (2021) [37]	Dengtai ye granules * (Yunnan Botanical Pharmaceutical Co., Ltd. (Kunming, China), Z53020312) *Folium Wrightiae Laevis*	<2 yr: 1 pack 2–5 yr: 3/2 pack >5 yr: 3 pack	3 times
Zhang (2022) [38]	Jiawei weijing decoction *Imperatae Rhizoma* 30 g, *Phragmitis Rhizoma* 15 g, *Stemonae Radix* 10 g, P*aeoniae Radix* 10 g, *Persicae Semen* 10 g, *Coicis Semen* 6 g, *Benincasae Semen* 6 g, *Armeniacae Semen* 6 g, *Perillae Fructus* 6 g, *Peucedani Radix* 6 g, *Plantaginis Semen* 6 g, *Pinelliae Tuber* 3 g, *Glycyrrhizae Radix et Rhizoma* 3 g, *Scorpio* 2 g	NR	2 times
Zhang (2023) [39]	Xiaoer Feike granules * (Changchun People’s Pharmaceutical Group Co., Ltd. (Changchun, China), Z20027415) *Ginseng radix et rhizoma*, *Poria*, *Atractylodis Macrocephalae rhizoma*, *Citri Unshius Pericarpium*, *Rhei radix et rhizome*, *Lycii Radicis Cortex*, *Glehniae radix*, *Glycyrrhizae Radix et Rhizoma*, *Artemisiae annuae herba*, *Ophiopogonis radix*, *Cinnamomic ramulus*, *Zingiberis Rhizoma Recens*, *Aconiti radix*, *Trichosanthis Fructus*, *Farfarae flos*, *Asteris radix et rhizoma*, *Mori Cortex Radicis*, *Astragali radix, Lycii fructus*, *Arisaema cum Bile*, *Galli gigerii endothelium corneum*, *Trionycis carapax*	<1 yr: 6 g 1–2 yr: 9 g	3 times
Zhi (2021) [40]	Dengtai ye granules * (Yunnan Botanical Pharmaceutical Co., Ltd. (Kunming, China), Z53020312) *Folium Wrightiae Laevis*	NR	3 times

NR, not reported; m, month; yr, year; *, There is no explanation for the capacity.

**Table 3:** GRADE certainty of evidence.

Outcomes	Subgroup	No. Participants (Studies)	Anticipated Absolute Effects (95% CI)	Relative Effect (95% CI)	Heterogeneity (*I*^2^)	Quality of Evidence
			Risk with Intervention Group			
TER	Total	1888 (22 RCTs)	159 more per 1000 (128 more to 191 more)	RR 1.20 (1.16 to 1.24)	0	⨁⨁⨁◯ Moderate ^a,^
Disappearance time of main symptom (spastic cough) (day)	Total	815 (9 RCTs)	MD 3.31 lower (3.51 lower to 3.11 lower)	-	94	⨁⨁⨁◯ Moderate ^a,^
	DY	560 (5 RCTs)	MD 3.16 lower (3.4 lower to 2.93 lower)	-	96	⨁⨁⨁◯ Moderate ^a,^
	WJ	92 (1 RCT)	MD 5.3 lower (6.17 lower to 4.43 lower)	-	Not applicable	⨁⨁◯◯ Low ^a,b^
	Sosiho decoction and Sabaek powder	68 (1 RCT)	MD 2.98 lower (3.77 lower to 2.18 lower)	-	Not applicable	⨁◯◯◯ Very Low ^a,b^
	Canyu dunke prescription	50 (1 RCT)	MD 5.08 lower (6.06 lower to 4.1 lower)	-	Not applicable	⨁◯◯◯ Very Low ^a,b^
	Zhenhai hwadam prescription	45 (1 RCT)	MD 2.58 lower (3.34 lower to 1.82 lower)	-	Not applicable	⨁⨁◯◯ Low ^a,b^
Recovery time of blood routine to normal range (day)	Total	472 (6 RCTs)	MD 2.79 lower (3.06 lower to 2.52 lower)	-	87	⨁⨁⨁◯ Moderate ^a^
	SB	352 (5 RCTs)	MD 2.58 lower (2.9 lower to 2.27 lower)	-	88	⨁⨁◯◯ Low ^a,b^
	DY	120 (1 RCT)	MD 3.28 lower (3.77 lower to 2.79 lower)		Not applicable	⨁⨁◯◯ Low ^a,b^
Hospitalization time (day)	Total	703 (9 RCTs)	MD 2.61 lower (2.85 lower to 2.38 lower)	-	83	⨁⨁⨁◯ Moderate ^a^
	SB	432 (6 RCTs)	MD 3.07 lower (3.37 lower to 2.78 lower)	-	39	⨁⨁⨁◯ Moderate ^a^
	DY	120 (1 RCT)	MD 3.73 lower (4.88 lower to 2.58 lower)	-	Not applicable	⨁⨁◯◯ Low ^a,b^
	Zhenhai hwadam prescription	83 (1 RCT)	MD 1.73 lower (2.25 lower to 1.21 lower)	-	Not applicable	⨁⨁◯◯ Low ^a,b^
	Sosiho decoction and Sabaek powder	68 (1 RCT)	MD 1.21 lower (1.9 lower to 0.51 lower)	-	Not applicable	⨁◯◯◯ Very Low ^a,b^

^a^ The overall bias is unclear; ^b^ The sample size did not meet the OIS criterion; CI, confidence interval; TER, total effective rate; RCT, randomized controlled trial; RR, risk ratio; MD, mean difference; SB, Sangbaipi decoction; DY, Dengtai ye granule; and WJ, Jiawei weijing decoction; More ⊕ indicates better quality of evidence.

## Data Availability

All data analyzed in this study are included in this published article and Supplementary Materials.

## Supplementary Materials

The following supporting information can be downloaded at: https://www.mdpi.com/article/10.3390/healthcare13101131/s1, Table S1: PRISMA 2020 checklist; Table S2: Search strategy for each database; Table S3: Frequency of herb; Table S4: Conventional and basic treatments; Table S5: Outcome measurement and result (*p*-value); Table S6: Sensitivity analysis of TER.

## Abbreviations

The following abbreviations are used in this manuscript:

CI	confidence interval
DY	Dengtai ye granule
GRADE	Grading of Recommendations Assessment, Development, and Evaluation
HM	herbal medicine
MD	mean difference
PLS	pertussis-like syndrome
RCTs	randomized clinical trials
ROB	risk of bias
RR	risk ratio
SB	Sangbaipi decoction
SMD	standardized mean difference
TER	total effective rate
WJ	Weijing decoction
WM	Western medicine
