# Supplementary material for: Efficacy and Safety of Combined Treatment with Traditional Herbal Medicine and Western Medicine for Children with Pertussis-like Syndrome: Systematic Review and Meta-Analysis
Source: Healthcare (Basel). 2025 May 13;13(10):1131. doi: 10.3390/healthcare13101131 (PMC12111028; doi:10.3390/healthcare13101131)
Supplement: Supplementary file 1 [file healthcare-13-01131-s001.zip › Supplementary Table S2. Search strategy used in each database.pdf]

Supplementary Table S2. Search strategy used in each database.

Medline via PubMed

	Searches	Results
#1	whooping cough[MeSH Terms]) OR (100-day cough[Title/Abstract]) OR (whooping cough[Title/Abstract]) OR (pertussis[Title/Abstract])	32,521
#2	Plants, Medicinal[MeSH Terms]) OR (Drugs, Chinese Herbal[MeSH Terms]) OR (Medicine, Chinese Traditional[MeSH Terms]) OR (Medicine, Kampo[MeSH Terms]) OR (Medicine, Korean Traditional[MeSH Terms]) OR (Herbal Medicine[MeSH Terms]) OR (Prescription Drugs[MeSH Terms]) OR (traditional Korean medicine[Title/Abstract]) OR (traditional Chinese medicine[Title/Abstract]) OR (traditional oriental medicine[Title/Abstract]) OR (Kampo medicine[Title/Abstract]) OR (herb*[Title/Abstract]) OR (decoction*[Title/Abstract]) OR (botanic*[Title/Abstract])	428,714
#3	#1 AND #2	189

EMBASE

	Searches	Results
#1	'whooping cough'/exp OR 'whooping cough' OR (whooping AND ('cough'/exp OR cough)) OR pertussis OR 'pertussis'/exp OR ('100 day' AND ('cough'/exp OR cough)) OR '100-day cough'	50,708
#2	'medicinal plant'/exp OR 'medicinal plant' OR 'chinese medicine'/exp OR 'chinese medicine' OR 'kampo medicine'/exp OR 'kampo medicine' OR 'kampo medicine (drug)'/exp OR 'kampo medicine (drug)' OR 'korean medicine'/exp OR 'korean medicine' OR 'herbal medicine'/exp OR 'herbal medicine' OR 'prescription drug'/exp OR 'prescription drug' OR 'oriental medicine'/exp OR 'oriental medicine' OR 'herb'/exp OR 'herb' OR 'decoction'	685,269
#3	#1 AND #2	413

CENTRAL

	Searches	Results
#1	MeSH descriptor: [whooping cough] explode all trees	10,707
#2	("whooping cough" OR "100-day cough" OR "pertussis"):ti,ab,kw	31,800
#3	MeSH descriptor: [Plants, Medicinal] explode all trees	92,861
#4	MeSH descriptor: [Medicine, Chinese Traditional] explode all trees	167,615
#5	MeSH descriptor: [Medicine, Kampo] explode all trees	2123
#6	MeSH descriptor: [Medicine, Korean Traditional] explode all trees	3588
#7	MeSH descriptor: [Herbal Medicine] explode all trees	277,631
#8	MeSH descriptor: [Prescription Drugs] explode all trees	37,120
#9	("traditional Korean medicine" OR "traditional Chinese medicine" OR "Traditional oriental medicine" OR "Kampo medicine" OR herb* OR decoction* OR botanic*):ti,ab,kw	200,533
#10	(#1 OR #2) AND (#3 OR #4 OR #5 OR #6 OR #7 OR #8 OR #9 OR #10) in Trials	2

CNKI

#1	(SU='百日咳'+'类百日咳'+'百日咳综合征') and (SU='中药'+'药'+'汤'+'丸'+'散'+'方'+'颗粒'+'胶囊'+'自拟')	1401

Wan Fang Database

#1	(Title=("百日咳"OR"类百日咳"OR"百日咳综合征") AND Title=("中药"OR"药"OR"汤"OR"丸"OR"散"OR"方"OR"颗粒"OR"胶囊"OR"自拟"))	505

	Chinese Scientific Journal Database (VIP)	
#1	(M=(百日咳 OR 类百日咳 OR 百日咳综合征) AND M=(中药 OR 药 OR 汤 OR 丸 OR 散 OR 方 OR 颗粒 OR 胶囊 OR 自拟))	167

	Oriental Medicine Advanced Searching Integrated System (OASIS)	
#1	(백일해 OR whooping cough OR pertissus) AND 한약	1

	Korean studies Information Service System (KISS)	
#1	(백일해 OR whooping cough OR pertissus) AND 한약	74

	Korea Citation Index (KCI)	
#1	(백일해 OR whooping cough OR pertissus) AND 한약	20

	Research Information Sharing Service (RISS)	
#1	전체 : 백일해 전체 : whooping cough 전체 : pertussis 전체 : 한약	4

	Citation Information by NI (CiNii)	
#1	(“百日咳” OR "百日咳综合征 " OR “whooping cough” OR “pertussis”) AND (“traditional Korean medicine” OR “traditional Chinese medicine” OR “Traditional oriental medicine” OR “Kampo medicine” OR “herb” OR “decoction” OR “b otanic” OR “漢方薬” OR “ハーブ” OR “散” OR “汤” OR “丸”)	64
