# Supplementary material for: Efficacy and Safety of Combined Treatment with Traditional Herbal Medicine and Western Medicine for Children with Pertussis-like Syndrome: Systematic Review and Meta-Analysis
Source: Healthcare (Basel). 2025 May 13;13(10):1131. doi: 10.3390/healthcare13101131 (PMC12111028; doi:10.3390/healthcare13101131)
Supplement: Supplementary file 1 [file healthcare-13-01131-s001.zip › Supplementary Table S3. Frequency of herb.pdf]

Supplementary Table S3. Frequency of herb.

Frequency	Herb
16	*Glycyrrhizae Radix et Rhizoma*
13	*Armeniacae Semen*
10	*Mori Cortex Radicis*
9	*Pinelliae Rhizoma, Perillae Fructus*
8	*Lepidii seu Descurainiae Semen, Scutellariae Radix, Fritillariae Thunbergii Bulbus, Persicae Semen*
6	*Stemonae Radix*
5	*Folium Wrightiae Laevis*
4	*Scorpio, Pinelliae Tuber, Lycii Radicis Cortex*
3	*Lumbricus, Batryticatus Bombyx, Zingiberis Rhizoma Recens, Coicis Semen, Phragmitis Rhizoma, Indigo Pulverata Levis, Ephedrae Herba, Benincasae Semen,*
2	*Fritillariae Cirrhosae Bulbus, Cicadidae Periostracum, Bupleuri Radix, Lonicerae Flos, Gypsum Fibrosum, Asiasari Radix et Rhizoma, Arctii Fructus, Belamcandae Rhizoma, Trichosanthis Radix, Paeoniae Radix, Peucedani Radix, Plantaginis Semen, Citri Unshius Pericarpium, Asteris Radix et Rhizoma, Farfarae Flos, Poria, Meretricis Concha*
1	*Gardeniae Fructus, Imperatae Rhizoma, Ginkgonis Semen, Syzygii Flos, Aucklandiae Radix, Aconiti Lateralis Radix Preparata, Aconiti Koreani Tuber, Gastrodiae Rhizoma, Gardeniae Fructus, Trichosanthis Pericarpium, Brassicae Semen, Evodiae Fructus, Codonopsis Pilosulae Radix, Eriobotryae Folium, Aurantii Fructus Immaturus, Artificial Bovis Calculus, Forsythiae Fructus, Platycodonis Radix, Schizonepetae Spica, Saposhnikoviae Radix, Ilicis pubescentis Radix, Ficus hirta vahl., Tribuli Fructus, Aristolochiae Fructus, Paridis Rhizoma, Ginseng radix et rhizoma, Atractylodis Macrocephalae rhizoma, Rhei radix et rhizome, Glehniae radix, Artemisiae annuae herba, Ophiopogonis radix, Cinnamomic ramulus, Aconiti radix, Trichosanthis Fructus , Astragali radix, Lycii fructus, Arisaema cum Bile, Galli gigerii endothelium corneum, Trionycis carapax*
