# Supplementary material for: Efficacy and Safety of Combined Treatment with Traditional Herbal Medicine and Western Medicine for Children with Pertussis-like Syndrome: Systematic Review and Meta-Analysis
Source: Healthcare (Basel). 2025 May 13;13(10):1131. doi: 10.3390/healthcare13101131 (PMC12111028; doi:10.3390/healthcare13101131)
Supplement: Supplementary file 1 [file healthcare-13-01131-s001.zip › Supplementary Table S4. Conventional treatments.pdf]

Supplementary Table S4. Conventional treatments

First author (year)	Conventional treatments
Li (2018) [22]	(1) Inhalation therapy<br>(2) Magnesium sulfate injection
Liu (2016) [23]	(1) Respiratory support (nasal catheter use, continuous positive airway pressure oxygen therapy)<br>(2) Antispasmodic treatments
Wang (2021) [30]	(1) Inhalation therapy<br>(2) Antispasmodic treatments<br>(3) Relieving cough symptoms
Zhang (2020) [36]	(1) Inhalation therapy<br>(2) Antispasmodic treatments<br>(3) Relieving cough symptoms
