# Supplementary material for: Efficacy and Safety of Combined Treatment with Traditional Herbal Medicine and Western Medicine for Children with Pertussis-like Syndrome: Systematic Review and Meta-Analysis
Source: Healthcare (Basel). 2025 May 13;13(10):1131. doi: 10.3390/healthcare13101131 (PMC12111028; doi:10.3390/healthcare13101131)
Supplement: Supplementary file 1 [file healthcare-13-01131-s001.zip › Supplementary Table S5. outcomes and p value.pdf]

Supplementary Table S5. Outcome measurement & Result (*p* -value).

First author (year)	Outcome measurement	Result (*p* -value)	Adverse Effect (*p* -value)
Chang	(1) TER	(1) E > C*: [95.0% vs 65.0%]	NR
(2019)	(2) Inflammatory factor level: WBC count	(2) E < C*	
[18]	(3) Inflammatory factor level: CRP level	(3) E < C*	
	(4) Pulmonary function index: RR	(4) E < C*	
	(5) Pulmonary function index: VT	(5) E > C*	
	(6) Pulmonary function index: TI/TE	(6) E > C*	
	(7) Disappearance time of main symptoms and pulmonary signs	(7) E < C*	
	(8) Recovery time of blood routine to normal range	(8) E < C*	
	(9) Hospitalization time	(9) E < C*	
Cui	(1) TER	(1) E > C+: [96.55% vs 86.21%]	NR
(2015)	(2) Disappearance time of main symptoms and pulmonary signs	(2) E < C*	
[19]	(3) Recovery time of blood routine to normal range	(3) E < C*	
	(4) Hospitalization time	(4) E < C*	
Cui	(1) TER	(1) E > C*: [97.5% vs 82.5%]	NR
(2019)	(2) Inflammatory factor level: CRP level	(2) E < C*	
[20]	(3) Disappearance time of main symptoms and pulmonary signs	(3) E < C*	
	(4) Hospitalization time	(4) E < C*	
Dong	(1) TER	(1) E > C*: [96.0% vs 82.0%]	NR
(2021)	(2) Recovery time of blood routine to normal range	(2) E < C^	
[21]	(3) Hospitalization time	(3) E < C^	
Li	(1) TER	(1) E > C*: [97.06% vs 85.29%]	NR
(2018)	(2) Disappearance time of main symptoms and pulmonary signs: spastic cough	(2) E < C*	
[22]	(3) Disappearance time of main symptoms and pulmonary signs: lung rhonchus sound	(3) E < C*	
	(4) Hospitalization time	(4) E < C*	
Liu	(1) Number of sputum suctions time	(1) E < C+	NR
(2016)	(2) Disappearance time of main symptoms and pulmonary signs: spastic cough	(2) E < C+	
[23]	(3) Disappearance time of main symptoms and pulmonary signs: wheezing and wet chirping sound	(3) E < C+	
	(4) Inflammatory factor level: CRP level	(4) E < C+	
Lu	(1) TER	(1) E > C*: [94.55% vs 81.1%]	E < C†:
(2022)	(2) Symptom scores	(2) E < C*	[5.45% vs 10.91%]
[24]	(3) Pulmonary function index: VT/kg (ml/kg), TI/TE, TPTEF/TE, VPEF/VE (%)	(3) E > C*	
	(4) Pulmonary function index: RR	(4) E < C*	
	(5) Inflammatory factor level: WBC count	(5) E < C*	

	(6) Inflammatory factor level: IgG, IgM	(6) E > C*	
Ren (2017) [25]	(1) TER<br>(2) Disappearance time of main symptoms and pulmonary signs<br>(3) Hospitalization time<br>(4) Recovery time of blood routine to normal range<br>(5) Symptom scores	(1) E > C*: [95.74% vs 82.98%]<br>(2) E < C*<br>(3) E < C*<br>(4) E < C*<br>(5) E < C*	NR
Tang (2020) [26]	(1) TER	(1) E > C*: [96.7% vs 73.3%]	NR
Tao (2020) [27]	(1) TER<br>(2) Inflammatory factor level: Lymphocyte percentage<br>(3) Symptom scores: main<br>(4) Symptom scores: improving vomiting, asthma and pulse condition<br>(5) Symptom scores: the number and duration of spasticity, shortness of breath, lingual frenulum ulcer<br>(6) Symptom scores: total<br>(7) Recurrence rate	(1) E > C*: [96.67% vs 76.67%]<br>(2) E < C†<br>(3) E < C*<br>(4) E < C*<br>(5) E < C†<br>(6) E < C*<br>(7) E < C†	E < C†: [4 cases of abdominal pain, diarrhea vs 5 cases of abdominal pain]
Wang (2019a) [28]	(1) TER<br>(2) Disappearance time of main symptoms and pulmonary signs	(1) E > C* : [95.65% vs 71.11%]<br>(2) E < C*	NR
Wang (2019b) [29]	(1) TER<br>(2) Disappearance time of main symptoms and pulmonary signs: spastic cough<br>(3) Disappearance time of main symptoms and pulmonary signs: lung rhonchus sound<br>(4) Hospitalization time<br>(5) Symptom scores	(1) E > C*: [97.62% vs 80.49%]<br>(2) E < C*<br>(3) E < C*<br>(4) E < C*<br>(5) E < C*	NR
Wang (2021) [30]	(1) TER<br>(2) Disappearance time of main symptoms and pulmonary signs: spastic cough<br>(3) Disappearance time of main symptoms and pulmonary signs: panting<br>(4) Disappearance time of main symptoms and pulmonary signs: shortness of breath<br>(5) Disappearance time of main symptoms and pulmonary signs: lung rhonchus sound	(1) E > C*: [93.02% vs 76.74%]<br>(2) E < C*<br>(3) E < C*<br>(4) E < C*<br>(5) E < C*	E < C#: [2.33% vs 4.65%]
Wang (2024) [31]	(1) TER<br>(2) Pulmonary function index: VT/kg, peak-to-volume ratio and peak-to-time ratio<br>(3) Inflammatory factor level: serum levels of IL-17, IL-6 and CRP	(1) E > C*: [95.92% vs 81.63%]<br>(2) E > C*<br>(3) E < C*	E < C: [4.08% vs 8.16%]
Yan (2019) [32]	(1) TER<br>(2) Disappearance time of main symptoms and pulmonary signs: spastic cough<br>(3) Disappearance time of main symptoms and pulmonary signs: lung rhonchus sound	(1) E > C*: [97.83% vs 82.61%]<br>(2) E < C*<br>(3) E < C*	NR
Zhang (2018a) [33]	(1) TER	(1) E > C*: [93.75% vs 71.88%]	NR

Zhang (2018b) [34]	(1) TER<br>(2) Disappearance time of main symptoms and pulmonary signs<br>(3) Recovery time of blood routine to normal range<br>(4) Hospitalization time	(1) E > C*: [96.67% vs 73.33%]<br>(2) E < C*<br>(3) E < C*<br>(4) E < C*	NR
Zhang (2019) [35]	(1) TER<br>(2) Disappearance time of main symptoms and pulmonary signs: spastic cough<br>(3) Disappearance time of main symptoms and pulmonary signs: panting<br>(4) Disappearance time of main symptoms and pulmonary signs: shortness of breath<br>(5) Disappearance time of main symptoms and pulmonary signs: lung rhonchus sound	(1) E > C* : [94.3% vs 80.0%]<br>(2) E < C*<br>(3) E < C*<br>(4) E < C*<br>(5) E < C*	None
Zhang (2020) [36]	(1) TER<br>(2) Disappearance time of main symptoms and pulmonary signs: spastic cough<br>(3) Disappearance time of main symptoms and pulmonary signs: shortness of breath<br>(4) Disappearance time of main symptoms and pulmonary signs: lung rhonchus sound<br>(5) Hospitalization time<br>(6) Inflammatory factor level: IL-6, IL-4, IFN-γ, TNF-α<br>(7) Cellular immune function: CD3+, CD4+, CD4+/CD8+<br>(8) Cellular immune function: CD8+	(1) E > C* : [95.00% vs 78.33%]<br>(2) E < C*<br>(3) E < C*<br>(4) E < C*<br>(5) E < C*<br>(6) E < C*<br>(7) E < C*<br>(8) E > C*	E < C*:<br>[5.00% vs 20.00%]
Zhang (2021) [37]	(1) TER<br>(2) Disappearance time of main symptoms and pulmonary signs: spastic cough<br>(3) Disappearance time of main symptoms and pulmonary signs: panting<br>(4) Disappearance time of main symptoms and pulmonary signs: shortness of breath<br>(5) Disappearance time of main symptoms and pulmonary signs: lung rhonchus sound<br>(6) Cellular immune function: Treg, TGF-β<br>(7) Cellular immune function: Th17/Treg, IL-17	(1) E > C*: [95.74% vs 91.49%]<br>(2) E < C*<br>(3) E < C*<br>(4) E < C*<br>(5) E < C*<br>(6) E > C*<br>(7) E < C*	E < C†:<br>[8.51% vs 10.64%]
Zhang (2022) [38]	(1) TER<br>(2) Symptom scores<br>(3) Pulmonary function index: VT, TI/TE, TPTEF/TE, VPEF/VE<br>(4) Pulmonary function index: RR, Rrs<br>(5) Inflammatory factor level: IL-4, IL-6, IFN-γ, TNF-α	(1) E > C*: [95.24% vs 80.95%]<br>(2) E < C+<br>(3) E > C+<br>(4) E < C+<br>(5) E < C+	E > C†:<br>[11.90% vs 9.53%]
Zhang (2023) [39]	(1) TER<br>(2) Pulmonary function index: VPEF/VE, TPTEF/TE, TV<br>(3) Pulmonary function index: RR<br>(4) Cellular immune function: CD3+, CD4+, CD4+/CD8+<br>(5) Cellular immune function: CD8+	(1) E > C*: [93.02% vs 76.74%]<br>(2) E > C*<br>(3) E < C*<br>(4) E > C*<br>(5) E < C†	NR
Zhi (2021) [40]	(1) TER<br>(2) Disappearance time of main symptoms and pulmonary signs: spastic cough<br>(3) Disappearance time of main symptoms and pulmonary signs: spastic cough attack<br>(4) Disappearance time of main symptoms and pulmonary signs: lung rhonchus sound<br>(5) Recovery time of blood routine to normal range	(1) E > C*: [95.0% vs 71.7%]<br>(2) E < C*<br>(3) E < C*<br>(4) E < C*<br>(5) E < C*	E > C†:<br>[4 cases of diarrhea vs 3 cases of diarrhea]

*, P<0.05; +, P<0.01; †, P>0.05; ^,P=0.000; #, P=1.000; E, experimental; C, control; NR, not reported; TER, total effective rate; WBC, white blood cells, CRP, C-reactive protein; RR, respiratory rate; VT, tidal volume; TI, inspiratory time; TE, expiratory time; TPTEF, time to reach peak tidal expiratory flow; VPEF, volume of air at the peak expiratory flow; VE, total expiratory volume; IG, immunoglobulin; IL, interlukin; IFN, interferon; TNF, tumor necrosis factor; CD, cluster of differentiation; Treg, regulatory T cells; Th, T helper cells; TGF, transforming growth factor; Rrs, total respiratory resistance.
