# Supplementary material for: Efficacy and Safety of Combined Treatment with Traditional Herbal Medicine and Western Medicine for Children with Pertussis-like Syndrome: Systematic Review and Meta-Analysis
Source: Healthcare (Basel). 2025 May 13;13(10):1131. doi: 10.3390/healthcare13101131 (PMC12111028; doi:10.3390/healthcare13101131)
Supplement: Supplementary file 1 [file healthcare-13-01131-s001.zip › Supplementary Table S6. Sensitivity analysis for TER.pdf]

Supplementary Table S6. Sensitivity analysis for total effective rate.

Excluding study	RR	95%CI	I^2 (%)	*p*-value
Chang 2019 [18]	1.19	1.15,1.24	0	<0.00001
Cui 2015 [19]	1.20	1.16,1.24	0	<0.00001
Cui 2019 [20]	1.20	1.16,1.24	0	<0.00001
Dong 2021 [21]	1.20	1.16,1.24	0	<0.00001
Li 2018 [22]	1.20	1.16,1.24	0	<0.00001
Lu 2022 [24]	1.21	1.16,1.25	0	<0.00001
Ren 2017 [25]	1.20	1.16,1.24	0	<0.00001
Tang 2020 [26]	1.19	1.15,1.24	0	<0.00001
Tao 2020 [27]	1.20	1.15,1.24	0	<0.00001
Wang 2019a [28]	1.19	1.15,1.23	0	<0.00001
Wang 2019b [29]	1.20	1.16,1.24	0	<0.00001
Wang 2021 [30]	1.20	1.16,1.24	0	<0.00001
Wang 2024 [31]	1.20	1.16,1.25	0	<0.00001
Yan 2019 [32]	1.20	1.16,1.24	0	<0.00001
Zhang 2018a [33]	1.19	1.15,1.24	0	<0.00001
Zhang 2018b [34]	1.19	1.15,1.24	0	<0.00001
Zhang 2019 [35]	1.20	1.16,1.24	0	<0.00001
Zhang 2020 [36]	1.20	1.15,1.24	0	<0.00001
Zhang 2021 [37]	1.21	1.16,1.25	0	<0.00001
Zhang 2022 [38]	1.20	1.16,1.24	0	<0.00001
Zhang 2023 [39]	1.20	1.16,1.24	0	<0.00001
Zhi 2021 [40]	1.19	1.15,1.23	0	<0.00001

RR, risk ratio; CI, confidence interval.
